# Instituting a Smoke-Free Policy for City Recreation Centers and Playgrounds, Philadelphia, Pennsylvania, 2010

**DOI:** 10.5888/pcd10.120294

**Published:** 2013-07-11

**Authors:** Raymond Leung, Giridhar Mallya, Lorraine T. Dean, Amna Rizvi, Leo Dignam, Donald F. Schwarz

**Affiliations:** Author Affiliations: Giridhar Mallya, Lorraine T. Dean, Amna Rizvi, Donald F. Schwarz, Philadelphia Department of Public Health, Philadelphia, Pennsylvania; Leo Dignam, Philadelphia Department of Parks and Recreation.

## Abstract

**Background:**

In the United States, more than 600 municipalities have smoke-free parks, and more than 100 have smoke-free beaches. Nevertheless, adoption of outdoor smoke-free policies has been slow in certain regions. Critical to widespread adoption is the sharing of knowledge about the policy development and implementation process. In this article, we describe our experience in making City of Philadelphia recreation centers and playgrounds smoke-free.

**Community Context:**

Of the 10 largest US cities, Philadelphia has among the highest rates of adult and youth smoking. Our objectives for an outdoor smoke-free policy included protecting against secondhand smoke, supporting a normative message that smoking is harmful, motivating smokers to quit, and mitigating tobacco-related sanitation costs.

**Methods:**

The Philadelphia Department of Public Health and the Department of Parks and Recreation engaged civic leaders, agency staff, and community stakeholders in the following steps: 1) making the policy case, 2) vetting policy options and engaging stakeholders, and 3) implementing policy. Near-term policy impacts were assessed through available data sources.

**Outcome:**

More than 220 recreation centers, playgrounds, and outdoor pools became smoke-free through a combined mayoral executive order and agency regulation. Support for the policy was high. Estimates suggest a policy reach of 3.6 million annual visitors and almost 850 acres of new smoke-free municipal property.

**Interpretation:**

Localities can successfully implement outdoor smoke-free policies with careful planning and execution. Such policies hold great potential for reducing exposure to secondhand smoke, promoting nonsmoking norms, and providing additional motivation for residents to quit smoking.

## Background

Health consequences of exposure to secondhand smoke are well documented (1). As these consequences have become better understood, government’s role has increased in affording protections from secondhand smoke, particularly through implementation of clean indoor air laws (2). Recently, outdoor smoke-free policies have also gained traction. As of December 2012, more than 600 municipalities have smoke-free parks and more than 100 have smoke-free beaches (3).

Nevertheless, adoption of outdoor smoke-free policies remains limited, particularly in large, urban areas outside of California. Thirteen of the 50 largest US cities (excluding Philadelphia) have smoke-free parks, but 7 are in California alone, and 6 are spread among the remaining 49 states and Puerto Rico (3). Widespread adoption is potentially being hindered by lengthy legislative processes, perceived enforcement challenges (4), and a lack of nearby communities with similar policies (5). Prior research highlights important strategies for smoke-free policy implementation, such as involving youth, using local data to garner support, and emphasizing community education; however, regulatory (rule-making) approaches may be underutilized (6).

In this article we describe our process for developing and implementing a smoke-free policy for municipal recreation centers and playgrounds in Philadelphia. We provide a legal framework emphasizing regulatory rule making and an implementation process that other municipalities can consider to advance similar policies. Finally, we summarize early policy impacts.

## Community Context

Of the 10 largest US cities, Philadelphia has the highest prevalence of adult smoking (Public Health Management Corporation [PHMC], Southeastern Pennsylvania Household Health Survey Data, 2008–2012) and among the highest rates of teen smoking (Centers for Disease Control and Prevention [CDC], Youth Risk Behavior Survey, 2011). Smoking-attributable diseases lead to approximately 2,100 deaths and $675 million in productivity losses annually in Philadelphia (7). Factors that account for these high rates include high levels of poverty, relatively low cigarette prices, ready availability of cigarettes, and smoking norms perpetuated by high rates of smoking (8).

In response to public health concerns surrounding tobacco, Philadelphia passed the Clean Indoor Air Worker Protection Law (CIAWPL) in 2006, which banned smoking in nearly all workplaces, restaurants, and bars as of January 2007. From 2008 through 2010, smoking among adults decreased from 27.3% to 25.2%, a statistically significant decline and the first large decrease in a decade (PHMC data, 2008–2012). This decrease may be partly attributable to CIAWPL and the federal cigarette tax increase in 2009. In 2009, the Philadelphia Department of Public Health (PDPH) developed a 5-year strategic plan to further decrease smoking and exposure to secondhand smoke.

In mid-2010, Philadelphia Mayor Michael Nutter requested that PDPH assess the public health evidence for outdoor smoke-free spaces and develop a smoke-free policy plan. Key areas of evidence in support of our policy included reducing risk of exposure to secondhand smoke, particularly for children, supporting a normative message that smoking is harmful, motivating smokers to quit, and mitigating sanitation costs associated with tobacco use. PDPH worked closely with Philadelphia Parks and Recreation (PPR) on this policy plan.

## Methods

We divided the policy change process into 3 stages: 1) making the policy case, 2) vetting policy options and engaging stakeholders, and 3) implementing policy, including communications and enforcement strategies (Table 1).

**Table 1:** Considerations in Developing a Smoke-Free Policy in Philadelphia; Questions and Issues for Each Locality

**Topic**	**Questions**
**Making the policy case**	What is the burden of smoking-related disease in your community? How would outdoor smoke-free policies decrease that burden? Are there particularly vulnerable or disparately affected populations that should be prioritized?
**Vetting policy options and engaging stakeholders**	Who are the key stakeholders and informants for developing and implementing a smoke-free policy? What are their main motivators and concerns? Which outdoor environments should be included in a smoke-free policy?
**Implementing policy, including communications and enforcement strategies**	Which policy enactment vehicle is most appropriate for your policy and municipality? How can you effectively promote awareness of the policy before, during, and after launch? What are achievable strategies for policy enforcement?

### Making the policy case

Establishing the public health evidence for policy change is critical to engaging diverse stakeholders with varied tobacco-related interests and for policy implementation and compliance (9). We outlined 4 main areas of evidence related to an outdoor smoke-free policy affecting municipal spaces. First, secondhand smoke can be harmful not only indoors but also in outdoor settings (10). Children, with their developing lungs, are particularly vulnerable to secondhand smoke (11), and in 2010, 23% of children in Philadelphia had been diagnosed with asthma (PHMC data, 2010).

Second, smoke-free policies support a normative message that smoking is unsafe and that nonsmokers have the right to be protected. This is especially significant for children because children are influenced by their perceptions of normal behavior (12). Clean indoor air laws are associated with lower odds of youth progressing from experimental to regular smoking (13). Accordingly, places that serve youth should be protected spaces for children.

Third, smoke-free policies are a strong motivator for smokers to try to quit. Smoke-free workplace policies are associated with increased cessation attempts (14). Because the City of Philadelphia employs 25,000 staff members and provides primary care to 90,000 patients through 8 community health centers, it stands much to gain from increased attempts to quit smoking and decreased smoking.

Fourth, outdoor smoke-free policies can alleviate sanitation burdens related to discarded cigarette butts and packages. Municipal cost savings are particularly important when localities are facing budget constraints. One analysis suggests that every pack of cigarettes leads to $0.22 in litter removal costs (15). This results in millions to tens of millions of dollars in costs annually for a large city.

### Vetting policy options and engaging stakeholders

Key internal and external stakeholders included 1) civic leaders, 2) PPR leadership and staff, and 3) community and public health advocates, including youth. Stakeholder engagement focused on identifying motivators for and concerns about outdoor smoke-free policies and deciding which spaces should be included in such policies.

Having championed passage of CIAWPL, the mayor knew of the tobacco control challenges in Philadelphia and the importance of smoke-free policies. A focus on child-serving institutions within government aligned with 1 of the mayor’s core objectives — improving children’s health and education. Thus, in mid-2010, he directed PDPH to explore outdoor smoke-free policy options with PPR leadership.

PDPH initiated a conversation with PPR in fall 2010. Discussion centered on the public health benefits of focusing on child-serving spaces and the potential challenges with compliance. PDPH had learned from other jurisdictions that signage, earned media, and staff and patron education could be sufficient for achieving compliance (4). PDPH and PPR discussed how these tasks could be shared across agencies and, as appropriate, with external partners.

**Figure 1:**
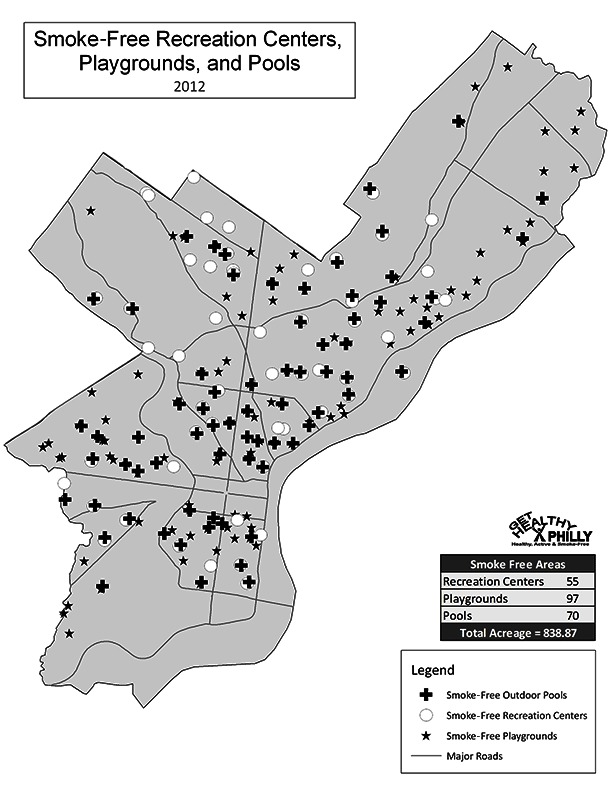
Map of smoke-free recreation centers, playgrounds, and pools in Philadelphia, 2012.

PPR oversees municipal recreation centers (n = 55), playgrounds (n = 97), pools (n = 70), and parks (n = 64) serving millions of visitors annually. The first 3 spaces are clustered in low-income neighborhoods and used primarily by children during after-school hours and by families on weekends (Figure 1). As in many large cities, recreation centers in Philadelphia are large complexes comprising athletic fields, playgrounds, buildings, and, in some cases, pools.

PDPH and PPR decided to focus on these child-serving spaces because they allowed for the strongest case to be made about public health benefits, mitigating harm from secondhand smoke, and creating nonsmoking norms. Additionally, parks presented numerous logistical barriers. Philadelphia’s parks system consists of 63 small neighborhood parks and Fairmount Park, an 8,000-acre park with vast green spaces, performance venues, museums, and historical buildings. PPR leadership noted that Fairmount Park’s size would make implementation in that setting particularly difficult. Moreover, neighborhood parks are represented by a decentralized network of local associations with varying degrees of influence, creating potential barriers to consensus development.

PPR leadership contacted 2 groups in late 2010 and early 2011 regarding a smoke-free policy for recreation spaces: on-the-ground agency staff and resident-led advisory councils. Key staff included 8 district managers, who oversee 10 to 15 recreation centers each. They expressed concerns similar to those of PPR leadership regarding enforcement, particularly in light of more pressing challenges such as violence and drug use on recreation center grounds. These issues were addressed in the educational sessions provided to PPR staff before roll-out. Advisory council leaders, who also have long-standing community ties to recreation centers, were generally supportive of the policy change. They identified a smoke-free policy as a way to protect children and perceived such policy as a natural extension of recreation center standards. Although accommodating adult smokers (both staff and patrons) was discussed, designated smoking areas were rejected because they would compromise the core goal of protecting children from the normative effects of seeing trusted adults smoking.

PDPH also engaged community and public health leaders outside of government through a variety of existing forums. The SmokeFree Philly Coalition brings together cessation providers, clinicians, employers, insurers, and public health organizations to promote cessation, decrease smoking initiation, and reduce secondhand smoke exposure among Philadelphians. The coalition recommended involving clean air and environmental organizations as allies, enhancing cessation support for municipal employees directly affected by the policy, and incorporating youth voices in the policy launch. To this point, the Philadelphia Youth Commission — comprising 15 youth commissioners appointed by the mayor and city council to represent the city’s youth — served as a critical partner. Commission members emphasized that teens are frequent users of recreation centers and pools even though most programming is oriented toward younger children. They also expressed interest in helping communicate the benefits of smoke-free spaces to peers, adults, and the media.

PDPH also presented evidence on the public health effects of smoke-free policies to the Get Healthy Philly Leadership Team, which is chaired by the mayor and includes leaders from private corporations, universities, health insurers, foundations, the School District of Philadelphia, and city council. Members viewed a smoke-free recreation policy as a natural extension of the city’s prior clean indoor air efforts and cited the new policy as motivation for other large employers to make their outdoor campuses smoke-free.

### Implementing policy

At the direction of the mayor, PDPH considered various vehicles for enacting the smoke-free policy (Table 2). Potential vehicles included legislation via city council, a regulation from a city agency, and an executive order of the mayor. PDPH leadership consulted with its legal counsel early in the process to vet these options because local policy vehicle options depend on local and state laws.

**Table 2:** Comparative Benefits and Costs of Policy Vehicles That Can Be Used to Advance an Outdoor Smoke-Free Policy^a^

Policy Vehicle	Pros	Cons
**Legislation**	Considered more representative of the people because legislators are directly accountable to them	Potentially a slower process; may not sufficiently engage the agency ultimately responsible for implementation and enforcement
	Efforts to undo the policy will require legislative action	
	Policy survives even if a successor administration does not support it	

**Administrative regulation (rule making)**	Potentially a quicker process	Administrative regulators are perceived as less accountable to the people because they are not elected
	Requires engagement and direct involvement of agency ultimately responsible for implementation and enforcement	Just as the policy may be quicker to adopt, it may also be quicker to undo by a successor administration

**Executive order**	Reinforces the direction from and support of the mayor; demonstrates a steadfast commitment to the issue by a “champion,” which is an important strategy in tobacco control (6).	Not enforceable with the general public
	Demonstrates a strong executive branch	
	Can be effectively used to garner more media exposure	

a Localities should consult with legal counsel early and often to ensure consideration of all possible policy options and vehicles. Local authorities may differ depending on jurisdiction.

Each policy vehicle has potential benefits and risks (Table 2). An executive order is a mayoral directive that affects management and operations of city government but lacks the full force of law. An executive order alone would not have been enforceable among the general public but can still be a powerful tool in commanding attention for an important issue. Legislation and regulation (rule making), on the other hand, carry the full force of law but each has pros and cons. A regulation is potentially quicker and involves the agency responsible for implementation; however, administrative regulators are perceived as less accountable because they are not elected, and a successor administration may quickly reverse a regulation. Legislation is potentially slower and may not sufficiently engage the agency responsible for implementation; nevertheless, legislators are directly accountable to the people, and efforts to reverse a policy will also require legislative action.

We targeted recreation centers and not parks more broadly, confirmed widespread stakeholder support, and focused on the public health benefits of protecting children from secondhand smoke. The mayor decided that these factors allowed the city to proceed with an executive order (directing the Parks and Recreation Commissioner to adopt a smoke-free policy) and PPR regulation (adopting a smoke-free policy). The executive order helped garner media attention and demonstrated the mayor’s strong commitment to this policy, and the regulation gave the policy the full force of law.

Once agreement was reached on the policy and the policy enactment vehicle, PDPH and SmokeFree Philly Coalition members began educating recreation center directors in early 2011. Seven sessions were conducted at meetings with individual district managers and the directors of the 10 to 15 recreation centers overseen by them. Education focused on the policy rationale; its applicability to all recreation centers, playgrounds, and pools; and how PPR staff should respond to noncompliance. Practical suggestions included pointing to a nearby smoke-free sign, providing a wallet card with information on cessation resources, and politely asking patrons to stop smoking. We believed that enforcement could be accomplished by educating patrons and encouraging self-enforcement, not by punishing or fining (4).

**Figure 2:**
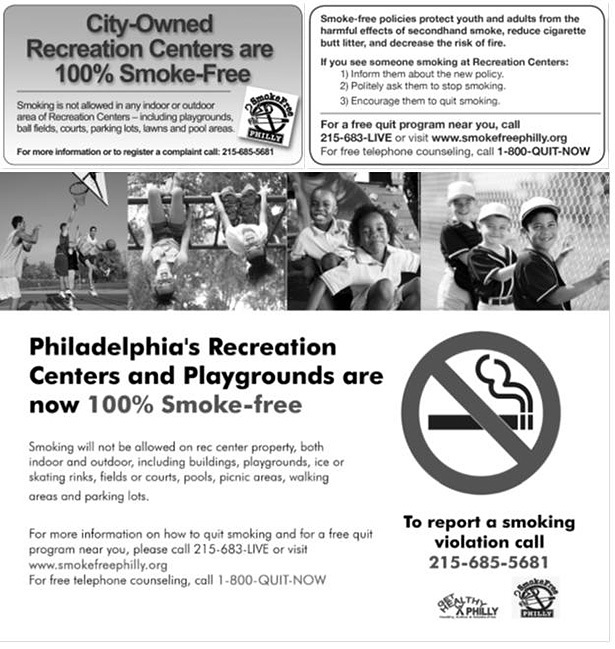
Smoke-free parks wallet card and poster, Philadelphia, Pennsylvania, 2011.

Recreation center leaders were provided with more than 4,000 wallet cards and 250 informational posters (Figure 2) for distribution and posting. PDPH developed a hotline through which staff and patrons could report violations of the policy. In response, PDPH educators would reach out to recreation center leaders to reinforce the policy and, as needed, provide in-person education for staff and patrons. PDPH distributed a memorandum explaining the policy to more than 400 sports leagues that hold permits for using PPR facilities. The memorandum was tailored to league organizers and coaches who could communicate the policy change to players and families.

Another key aspect of enforcement was facilitating compliance through cessation support because smoke-free policies can induce smokers to make quit attempts (14). Wallet cards, informational posters, and signage were meant to encourage people to call the Pennsylvania Free Quitline (1-800-QUIT-NOW) and visit a website (www.smokefreephilly.org). PDPH and PPR staff collaborated to offer free community-based cessation classes at recreation centers. Even before adopting an outdoor smoke-free policy, the City of Philadelphia expanded cessation benefits for 7,000 city employees and their dependents. PDPH partnered with local Medicaid plans to put Food and Drug Administration (FDA)-approved cessation therapies on their formularies at low to no cost for patients. PDPH implemented a large mass media campaign to promote quitting and sponsored a quitline-based nicotine patch giveaway in 2010 and 2011 for 10,000 Philadelphians.

In May 2011, Mayor Michael Nutter signed Executive Order 5–11 at Kingsessing Recreation Center. Attendees included Health and Parks and Recreation commissioners, a youth commissioner, and children from the neighborhood. We selected the location based on high rates of smoking in the area, and we facilitated youth involvement by selecting locations with child-oriented facilities (eg, swing sets), and proximity to a nearby school. This setting highlighted the policy’s public health potential to reduce secondhand smoke exposure of vulnerable populations and promote nonsmoking norms. Press, partners, and community members attended the event in response to a media advisory, a press release, and communications through the SmokeFree Philly Coalition.

In subsequent weeks, PDPH and SmokeFree Philly Coalition members continued educational sessions with recreation center leaders, sports league organizers and coaches, and other stakeholders. In July 2011, the Parks and Recreation commissioner promulgated a regulation making city recreation centers and playgrounds smoke-free. The regulation became effective in August 2011. During the next 6 months, more than 1,000 permanent smoke-free signs were installed at all recreation centers, playgrounds, and pools.

## Outcome

We used available data sources to estimate near-term effects of this policy, including PPR administrative data, geospatial analysis, community focus groups, and a telephone-based survey of Philadelphia smokers that had been fielded to evaluate a contemporaneous mass media campaign.

A total of 222 recreation centers, playgrounds, and pools have become smoke-free. This change added nearly 850 smoke-free acres to Philadelphia (Figure 1). An estimated 3.6 million annual visits — most of which are made by children — will now be smoke-free (Table 3). In addition, the *Philadelphia Inquirer, *the city’s main daily paper, published an editorial praising the policy (16).

**Table 3:** Estimated Population Impact^a^ by Site

Type of Site	Number of Sites	Average Visits/wk	Weeks/y	Visits/y
Recreation centers	55	27,249	52	1,416,948
Outdoor pools	70	118,278	8	946,224
Playgrounds	97	25,345	52	1,317,940
Total	222	NA^b^	NA^b^	3,681,112

a Based on the number of Parks and Recreation visitors in 2011.

b Outdoor pools are open only 8 weeks a year, so no total is given.

On the basis of coverage of the executive order signing ceremony in May 2011, we estimate that more than 770,000 impressions were generated via television and print media (230,000 and 541,518 impressions, respectively) (Terry Johnson, personal communication, June 2012). This figure does not include impressions for 5 major online media outlets for which data were unavailable. Television and online stories featured parents expressing gratitude that their children were being protected. According to a monthly cross-sectional monitoring survey of adult smokers in Philadelphia from June 2011 through September 2011, approximately 50% were aware of the smoke-free recreation center policy and, of those, more than 75% were supportive of it (Laura Gibson, Sarah Parvanta, Bob Hornik, Michelle Jeong, Emily Brennan; Annenberg School for Communication, campaign recall monitoring survey [unpublished raw data], 2011) (Table 4). PDPH, in partnership with WHYY (local public media) and the Penn Project for Civic Engagement (Penn Project), hosted 4 civic dialogue sessions in September and October 2011 entitled “Smoke Signals: Forums on Tobacco Use in Philadelphia.” The Penn Project sought volunteer participants from community groups and high schools, and more than 75 people participated in 4 sessions. Groups were asked by a moderator whether they supported 9 different policy proposals. Six of 7 groups at these sessions expressed support for protecting people from secondhand smoke in outdoor spaces.

**Table 4:** Awareness of and Support for Smoke-Free Policy^a^

Awareness and Support	June 2011	July 2011	August 2011	September 2011
Adult smokers aware of policy	61%	47%	51%	52%
Adult smokers supporting policy	76%	72%	74%	77%

a Based on the number of Philadelphia adult smokers surveyed between June and September 2011 who were aware of and supporting the smoke-free policy. Source: Laura Gibson, Sarah Parvanta, Bob Hornik, Michelle Jeong, Emily Brennan. Campaign recall monitoring survey [unpublished raw data]. Philadelphia (PA): Annenberg School for Communication; 2011.

## Interpretation

Smoke-free policies affecting outdoor municipal spaces hold great potential to reduce exposure to secondhand smoke, promote nonsmoking norms, and provide additional motivation to quit smoking. Our experience suggests that localities can successfully review and synthesize the evidence for such policies, engage stakeholders in policy assessment, and implement and enforce these policies to enhance the public’s health.

In Philadelphia, PDPH led efforts to document the benefits of an outdoor smoke-free policy in light of high smoking rates among adults and youth, normative smoking behaviors in low-income communities, and the large number of children who use municipal recreation spaces and are especially vulnerable to secondhand smoke. PDPH worked closely with PPR to vet policy options and focus initial efforts on facilities that primarily serve children. Critical input was solicited from city residents, community leaders, agency staff, and public health organizations.

We enacted our policy through administrative actions of the executive branch, which have been overlooked as a critical tobacco control tool. Depending on local context, other communities should consider this approach. By combining a mayoral executive order and an agency regulation, we were able to garner attention for our smoke-free outdoor policy, advance it quickly, and instill it with the full force of law. Lastly, we implemented a strong communications strategy for staff, patrons, and the public to ensure awareness of the policy change and engage community members in efforts toward compliance. These communications were also leveraged to promote evidence-based smoking cessation resources.

Our observations have several limitations. One, we did not evaluate compliance through objective measures. We focused on documenting the process of policy adoption as a model for other localities. Two, we were able to adopt our policy with backing by elected and appointed leaders. Although our legal approach (rule making instead of legislation) was appropriate for Philadelphia, each municipality should consult with legal counsel because local authorities may differ. Moreover, engaging legal counsel is important to ensure that localities are adhering to restrictions placed on the use of government funds. Three, we relied on policy, legal, analytic, and communications staff with tobacco control expertise throughout the policy development process. Not all localities may have these resources, though municipalities can rely on existing public health staff with similar capabilities. Despite these limitations, we believe our experience can guide other municipalities interested in an outdoor smoke-free policy.
